# Antidiabetic potential of *Gynura procumbens* (Lour.) Merr.: a review of *in vitro* and *in vivo* studies

**DOI:** 10.3389/fphar.2025.1646591

**Published:** 2025-08-14

**Authors:** Nurul Hafizah Mohd Nor, Farah Hanan Fathihah Jaffar, Mohd Izhar Ariff Mohd Kashim, Mohd Helmy Mokhtar

**Affiliations:** ^1^ Institute of Islam Hadhari, Universiti Kebangsaan Malaysia, Bangi, Selangor, Malaysia; ^2^ Department of Physiology, Faculty of Medicine, Universiti Kebangsaan Malaysia, Cheras, Kuala Lumpur, Malaysia; ^3^ Centre of Shariah, Faculty of Islamic Studies, Universiti Kebangsaan Malaysia, Bangi, Selangor, Malaysia

**Keywords:** diabetes mellitus, antidiabetic plant, Gynura procumbens, hypoglycaemic, antioxidative, phytochemical

## Abstract

Diabetes mellitus (DM) is a chronic metabolic disease that affects around 10.5% of adults worldwide. It leads to significant complications, including nephropathy, retinopathy and cardiovascular disease. Conventional treatments for DM often involve the long-term use of pharmacological agents, which can be costly and are associated with various side effects. Due to these challenges, there is growing interest in complementary treatments, particularly those derived from botanical drugs, to explore their potential antidiabetic properties. *Gynura procumbens* (Lour.) Merr. (GP) has been scientifically studied and shown to possess antioxidant properties that lead to a significant reduction in blood glucose levels and an improvement in lipid profile. The aim of this review is therefore to provide a detailed overview of the current state of knowledge on the antidiabetic potential of GP based on four *in vitro* studies and 12 *in vivo* studies. GP extract in concentrations between 50 mg and 3,000 mg shows promising potential as an antidiabetic agent, with some studies suggesting comparable efficacy to metformin in the treatment of diabetes. In addition, phytochemical studies of GP have revealed a diverse phytochemical metabolite, with a predominance of polyphenolic metabolites, especially phenolic acids and flavonoids, extracted from various solvents. However, the evidence remains mixed, as other studies have presented varying results on the efficacy of GP in the treatment of diabetes. This could be due to the lack of standardisation of the extract preparation, insufficient information on the bioactive metabolite responsible for the observed effects and the lack of clinical studies. Therefore, more comprehensive studies including clinical trials are needed to clarify the discrepancies in the findings and provide a clearer effect of GP in alleviating DM. With these improvements, GP could complement standard DM treatments and offer patients a safer, more holistic approach.

## 1 Introduction

Diabetes mellitus (DM) is a chronic metabolic disease characterised by persistent hyperglycaemia due to impaired insulin secretion, insulin action or both (Kharroubi and Darwish, 2015; Artasensi et al., 2020). It can be divided into type 1 DM (T1DM) and type 2 DM (T2DM). T1DM is characterised by an absolute insulin deficiency, which is often accompanied by symptoms such as thirst, weight loss and polyuria and accounts for 10% of DM cases (Sun et al., 2021). T2DM, on the other hand, is characterised by insulin resistance in the target tissue, relatively insufficient insulin secretion and resulting dysfunction of the β-cells, which often causes no symptoms (Nyenwe et al., 2011). The DM condition leads to long-term complications affecting various organs, including the eyes, kidneys, nerves, heart and blood vessels. There are several factors that can lead to DM, such as genetic inheritance, viral infections, unhealthy lifestyle and other physical or chemical damage that leads to the destruction of β-cells (Roglic, 2016).

The global prevalence of DM is increasing at an alarming rate and poses major challenges for healthcare systems worldwide (Hossain et al., 2024). A recent study highlights that the global prevalence of DM is estimated to reach 700 million people by 2045 if current trends continue, emphasising the urgent need for effective prevention and management strategies (Sun et al., 2022). Another study notes that the prevalence of DM is particularly high in low- and middle-income countries, where health systems are often less well equipped to cope with the disease burden (Dendup et al., 2018).

Currently, conventional treatments for DM mainly focus on controlling blood glucose levels through the use of medications such as insulin, metformin and other antidiabetic drugs (Weinberg Sibony et al., 2023). However, they often have side effects and do not address the multifactorial nature of the disease. For example, long-term use of certain medications can lead to gastrointestinal problems, weight gain and an increased risk of cardiovascular events (Shurrab and Arafa, 2020). In addition, these treatments do not adequately target the underlying causes of DM, such as insulin resistance and beta-cell dysfunction.

As a result, there is growing interest in alternative and complementary therapies, including herbal medicines, for the treatment of diabetes. Botanical drugs offer a holistic approach by targeting multiple metabolic pathways involved in the development of DM (Pang et al., 2019). They are often considered to have few side effects and may offer additional benefits such as antioxidant and anti-inflammatory effects (Adam et al., 2022). In addition, conventional antidiabetic drugs may be costly or insufficiently available in low- and middle-income countries. Botanical drug treatments, especially those grown locally, are often considered more accessible and affordable (Mohan et al., 2020; Chaachouay and Zidane, 2024). Furthermore, integrative medicine, which combines evidence-based complementary practises with conventional treatments, is becoming increasingly important in modern healthcare systems. This has led to increased research into the antidiabetic properties of various botanical drugs, which have shown promising results in both preclinical and clinical studies (Vivó-Barrachina et al., 2022).

Botanical drugs represent a vast and invaluable reservoir of bioactive metabolites, many of which have a wide range of pharmacological properties. Due to their diverse mechanisms of action and generally lower risk of adverse effects, they represent a promising avenue for research and hold significant potential for improving the comprehensive treatment of DM (Yedjou et al., 2023). *Gynura procumbens* (Lour.) Merr. (GP) is an herbal plant rich in bioactive metabolites that may provide therapeutic benefits for a number of diseases. It is a perennial evergreen shrub belonging to the Asteraceae family (Tan et al., 2016). It is called ‘Sambung Nyawa’ or Sabungai in Malay (Jobaer et al., 2023), ‘bai bing cha’ by Chinese and Malay communities (Murugaiyah et al., 2018), and ‘pyar-hmee’ in Myanmar (Aung et al., 2021). It is also known as ‘longevity spinach’ (Jobaer et al., 2023). This green vegetable is typically small-growing and reaches a height of around 1–3 m (Tan et al., 2016). It also has yellow, slender and panicle-shaped flower heads that are 1–1.5 cm long and ovate-elliptical or lanceolate leaves that are 3.5–8 cm long and 0.8–3.5 cm wide. This edible herbaceous plant is widely distributed in Malaysia, Indonesia, Thailand, Vietnam and China. In Thailand, the GP leaves are used as a popular vegetable for a variety of culinary creations and play an important role in dishes such as chilli paste, curries, salads and soups and can be enjoyed both raw and cooked (Kaewseejan et al., 2015). In Malaysia, it is often eaten uncooked with rice and used as an ingredient in salads and ulam (Hew and Gam, 2011).

This plant has long been used in traditional medicine for the treatment of diabetes, hyperlipidaemia, kidney disease, hypertension, fever, constipation, skin irritation, migraine, rheumatism, haemorrhoids and colon cancer (Zhang and Tan, 2000; Rosidah et al., 2009; Hassan et al., 2010; Mohamed et al., 2023). In addition, this plant has a variety of therapeutic properties, such as anti-hyperlipidaemic, anti-inflammatory, antibacterial, antifungal, antihypertensive, antioxidant and anticancer properties (Iskander et al., 2002; Kim et al., 2011; Algariri et al., 2013; Hew et al., 2013; Jarikasem et al., 2013; Jeon and Kwon, 2016). Since it is a natural-based product, it is a better option with minimal side effects compared to the commercially available synthetic drugs. The availability of GP at a reasonable price and easy accessibility makes it a significant alternative to modern medicine, which is important specifically for an underprivileged community. Furthermore, the increasing use of GP as a natural remedy has led to this plant having potential for commercial cultivation and processing of organic products due to the growing markets for herbal raw materials and processed products.

Therefore, this review aims to summarise the current information on the potential health benefits of GP in DM and its complications from animal models and *in vitro* studies. The traditional use of this plant in folk medicine combined with scientific validation emphasises its potential as a complementary therapy for the treatment of DM.

## 2 Nutritional and Phytochemistry of *Gynura procumbens* (Lour.) Merr

The nutritional analysis of GP leaves revealed that they contain 13.59% crude fibre and 16.92% ash. Its nutritional properties, especially the high fibre content and low glycaemic profile, increase the antidiabetic potential of GP and make it a promising candidate for dietary intervention for the prevention or treatment of T2DM (Nath et al., 2023). In addition, GP leaves contain a low composition of protein (6.2%), moisture (6.2%), carbohydrate (0.4%), and fat (0.07%) (Nath et al., 2023). This is important as it can help reduce the risk of various chronic diseases, such as type 2 diabetes by improving insulin sensitivity, regulating blood glucose levels, and reducing the risk of developing diabetes (Jobaer et al., 2023). The nutritional contents of the GP extract are listed in Table 1.

**TABLE 1:** Proximate nutritional contents of GP extract.

Component	Content	References
Proximate analysis (%)		Nath et al. (2023)
Ash	16.92	Nath et al. (2023)
Moisture	6.2	Nath et al. (2023)
Carbohydrate	0.4	Nath et al. (2023)
Protein	6.2	Nath et al. (2023)
Fat	0.07	Nath et al. (2023)
Crude fiber	13.59	Nath et al. (2023)

Meanwhile, phytochemical studies of GP have revealed a diverse phytochemical metabolite, with a predominance of polyphenolic metabolites, especially phenolic acids and flavonoids, extracted from various solvents. Of the phenolic acids, gallic acid, protocatechuic acid, p-hydroxybenzoic acid, vanillic acid and syringic acid, which belong to the hydroxybenzoic acid subclass were consistently detected. They can be detected in the aqueous, ethanolic and ethyl acetate fractions of the leaves (Kaewseejan et al., 2015; Liu et al., 2019). Caffeic acid, p-coumaric acid, ferulic acid and sinapic acid, which belong to the hydroxycinnamic acids, can be found in both polar (aqueous, methanol) and semi-polar (ethyl acetate) extracts, indicating their solubility in a range of solvents (Kaewseejan et al., 2015; Alam et al., 2016; Liu et al., 2019; Kim et al., 2021). In addition, phenolic acids have been shown to lower blood glucose levels and protect against chronic diseases caused by hyperglycaemia through antioxidant protection (Deka et al., 2022).

Important flavonoids such as rutin (a quercetin glycoside), quercetin, myrricetin, kaempferol, apigenin and luteolin have been found in several extracts, reflecting their richness and structural diversity within the plant matrix (Kaewseejan et al., 2015; Liu et al., 2019; Kim et al., 2021). These metabolites act on multiple diabetes targets and regulate key signalling pathways that improve both the symptoms and complications of T2DM (Dhanya, 2022). Furthermore, chlorogenic acid, a biologically active caffeoylquinic acid ester, was exclusively detected in the methanolic extract. This indicates its preferential solubility in more polar organic solvents (Kim et al., 2021).

In addition to polyphenols, the methanolic extract of GP also contained considerable amounts of non-phenolic bioactive metabolites, including coumarins such as oxypeucedanin and isoimperatorin, which are known for their anti-inflammatory and vasodilatory effects (Kim et al., 2021; Flores-Morales et al., 2023; Kubrak et al., 2025). Indole-derived compounds such as indole-3-carboxylic acid and kynurenic acid, a tryptophan metabolite with neuroprotective properties, have also been identified and broaden the pharmacological spectrum of the plant (Kim et al., 2021).

On the other hand, the methanolic extract of GP also contained numerous lipophilic metabolites. These include lutein, a potent antioxidant involved in chlorophyll biosynthesis and known for its antimicrobial properties. Several triterpenoids such as lupeol, β-amyrin and friedelanol acetate, which are associated with antidiabetic, anti-inflammatory, anticancer and hepatoprotective activities have also been detected in the methanolic extract of GP (Szakiel et al., 2012; Saha and Bandyopadhyay, 2020; Kim et al., 2021; Jobaer et al., 2023; Dalimunthe et al., 2024). In addition, phytosterols such as stigmasterol and a mixture of stigmasterol and β-sitosterol have also been identified, which are known for their cholesterol-lowering effect as well as their cytoprotective potential and reduced hyperglycaemic effects (Vezza et al., 2020; Jobaer et al., 2023). The antidiabetic effect of stigmasterol may be due to the regeneration of the pancreatic β-cells of Langerhans and thus the secretion of insulin, which controls blood glucose levels (Eidi et al., 2006; Nualkaew et al., 2015).

However, the non-specific bioactivity of natural products is often a hurdle in their bioassay evaluation, including GP (Baell, 2016). Some of its metabolites can behave as pan-assay interference compounds (PAINS), causing false positive signals in various assays and thus complicating their interpretation. For example, the pyrrolizidine alkaloids in GP are known to cause interference in a variety of bioassays due to their reactive nature and the formation of DNA adducts (Ji et al., 2019). Lupeol, stigmasterol and β-sitosterol can interfere with enzyme assays and receptor binding studies due to their structural similarity to endogenous steroids and thus bind non-selectively to other non-endogenous targets (Jobaer et al., 2023). In addition, polyphenolic metabolites such as chlorogenic acid, caffeic acid and various flavonoids could also interfere with redox-based assays (Kim et al., 2021). In addition, some pharmacological profiles of GP may also correspond to mechanisms typical of PAINS. For example, its anti-inflammatory activities have been shown to inhibit the NF-κB signalling pathway and downregulate the expression of pro-inflammatory cytokines such as IL-1β and TNF-α (Wong et al., 2015; Tan et al., 2022). These activities, which involve multiple signalling pathways, are often considered characteristic of PAINS.

Nonetheless, the potential for interference by PAINS does not undermine the medical value of GP. Evidence from a wide range of *in vitro* and *in vivo* studies, as well as from thousands of years of traditional use, has provided a solid basis for its efficacy and safety. This evaluation can be further enhanced by modern analytical and computational techniques. High-resolution techniques, in particular UHPLC-QTOF-MS/MS and HPLC-MS, and the use of computational tools such as molecular docking and molecular dynamics simulations enable the prediction of binding affinities and target interactions. These strategies are particularly useful for the identification of compounds with high promiscuity and thus potential PAINS properties (Tithi et al., 2023). By integrating such approaches, researchers can minimise concerns about assay interference and detect consistent, target-specific biological effects across multiple experimental platforms.

Table 2 provides an overview of the phytochemical metabolite found in GP.

**TABLE 2:** Phytochemical metabolites found in GP leaves.

Classification	Phytochemical metabolites	Extraction methods	References
Polyphenol	Gallic acid	Aqueous extract; ethanolic extract; ethyl acetate fraction	Liu et al. (2019)
Polyphenol	Protocatechuic acid	Aqueous extract; ethanolic extract; ethyl acetate fraction	Kaewseejan et al. (2015), Liu et al. (2019)
Polyphenol	*p*-Hydroxybenzoic acid	Ethanolic extract; ethyl acetate fraction	Kaewseejan et al. (2015)
Polyphenol	Vanillic acid	Ethanolic extract; ethyl acetate fraction	Kaewseejan et al. (2015)
Polyphenol	Syringic acid	Ethanolic extract; ethyl acetate fraction	Kaewseejan et al. (2015)
Polyphenol	Caffeic acid	Aqueous extract; ethanolic extract; ethyl acetate fraction; methanolic extract	Liu et al. (2019), Kim et al. (2021)
Polyphenol	*p*-coumaric acid	Aqueous extract; ethanolic extract; ethyl acetate fraction; methanolic extract	Liu et al. (2019), Kim et al. (2021)
Polyphenol	Ferulic acid	Ethanolic extract; ethyl acetate fraction	Kaewseejan et al. (2015)
Polyphenol	Sinapic acid	Ethanolic extract; ethyl acetate fraction	Kaewseejan et al. (2015)
Polyphenol	Rutin	Aqueous extract; ethanolic extract; ethyl acetate fraction	Kaewseejan et al. (2015), Liu et al. (2019)
Polyphenol	Myricetin	Aqueous extract; ethanolic extract; ethyl acetate fraction	Kaewseejan et al. (2015), Liu et al. (2019)
Polyphenol	Quercetin	Aqueous extract; ethanolic extract; ethyl acetate fraction	Kaewseejan et al. (2015), Liu et al. (2019)
Polyphenol	Apigenin	Aqueous extract; ethanolic extract; ethyl acetate fraction	Liu et al. (2019)
Polyphenol	Kaempferol	Aqueous extract; ethanolic extract; ethyl acetate fraction	Kaewseejan et al. (2015), Liu et al. (2019)
Polyphenol	Chlorogenic acid	Methanolic extract	Kim et al. (2021)
Coumarins	Oxypeucedanin	Methanolic extract	Kim et al. (2021)
Coumarins	Isoimperatorin	Methanolic extract	Kim et al. (2021)
Indolecarboxylic acids and derivatives	Indol-3-Carboxylic acid	Methanolic extract	Kim et al. (2021)
Tryptophan metabolite	Kynurenic acid	Methanolic extract	Kim et al. (2021)
Carotenoid	Lutein	Methanolic extract	Kim et al. (2021)
Polyphenol	Luteolin	Methanolic extract	Kim et al. (2021)
Diterpenoid	Phytol	Methanolic extract	Jobaer et al. (2023)
Triterpenoid	Lupeol	Methanolic extract	Jobaer et al. (2023)
Phytosterol	Stigmasterol	Methanolic extract	Jobaer et al. (2023)
Triterpenoid	Friedelanol acetate	Methanolic extract	Jobaer et al. (2023)
Triterpenoid	β-amyrin	Methanolic extract	Jobaer et al. (2023)
Phytosterol	Mixture of stigmasterol and β-sitosterol	Methanolic extract	Jobaer et al. (2023)

## 3 Pharmacokinetics of *Gynura procumbens* (Lour.) Merr

The pharmacokinetics of GP have significant implications for its potential therapeutic use; however, they are far from clear. The antidiabetic effect of GP, which is attributed to increased glucose uptake and improved insulin sensitivity, may also be associated with metabolic interactions (Hassan et al., 2010; Guo W. et al., 2021). There are also scants *in vivo* reports on the absorption, distribution, metabolism and excretion profiles of GP and its active fractions. However, useful information can be extrapolated from the known phytochemical profile of this plant and pharmacokinetic studies on structurally related metabolites.

It has been hypothesised that the absorption of polyphenolic metabolites extracted from GP may be poor. The flavonoids and phenolic acids generally have low oral bioavailability due to a high degree of first-pass metabolism (Xin et al., 2011; Ye et al., 2023). However, there is evidence that the presence of other molecules in GP can increase its absorption. For example, chlorogenic acid (CA), a predominant phenolic metabolite of GP, was found to increase intestinal absorption and improve bioavailability when combined with other phytochemicals in plants, with the improvement increasing from 6.7% (CA alone) to 16.0% (Wang et al., 2022). Pharmacokinetic analyses of the total extract are also not available, and the distribution of GP metabolites in the body remains unknown. However, similar flavonoids and phenolic acids have been found to accumulate in metabolically active organs such as adipose tissue, intestine, liver, kidneys and lungs (Ye et al., 2023). These results are in line with previous studies that have shown that GP molecules are widely distributed in numerous tissues, possibly leading to hepatoprotective and nephroprotective effects (Tahsin et al., 2022).

In addition, the metabolites of GP are generally expected to undergo phase I and phase II metabolism, namely, oxidative, reductive, hydrolytic, glucuronidated and sulphated, and methyl-mediated metabolic reactions, which mainly occur in the liver and gastrointestinal tract (Xin et al., 2011; Wang et al., 2022; Ye et al., 2023). Flavonoids and phenolic acids from GP are also likely to be metabolised via these pathways, leading to the formation of conjugated metabolites that can be detected in plasma after oral administration. Such modulating effects may also contribute to its antidiabetic effect. Although the excretion patterns of GP metabolites are not well studied, it is assumed that, like other plant polyphenols, they are mainly excreted via the kidneys and bile (Ye et al., 2023).

Overall, the data available to date indicate that the biological effects of GP are significantly influenced by its low bioavailability, extensive metabolism and wide tissue distribution. Further studies are needed to clarify this involvement and to translate the molecular understanding to the clinical level of GP-targeted therapy.

## 4 Effects of *Gynura procumbens* (Lour.) Merr. on diabetes mellitus

Previous studies have documented the potential antidiabetic effect of GP, which is characterised by a reduction in blood glucose levels and improved glucose tolerance in DM. A total of 16 studies were found including four *in vitro* studies and 12 *in vivo* studies.

### 4.1 *In vitro* studies

*In vitro* studies have elucidated the mechanisms underlying the antidiabetic properties of GP. The *in vitro* experiments revealed the mechanisms and signalling pathways at the molecular level involving a variety of cell types. For example, one study reported that the aqueous extract of GP triggered translocation of GLUT4 membrane into 3T3-L1 adipocytes, while both the aqueous and ethanolic extracts triggered translocation of GLUT4 membrane into C2C12 muscle cells, which is a critical site for insulin-stimulated glucose uptake (Merz and Thurmond, 2020; Aung et al., 2021). Interestingly, GP had a stronger effect on GLUT4 membrane translocation in C2C12 muscle cells compared to 3T3-L1 adipocyte cells. The observed effect could be due to the tissue-specific difference in sensitivity or responsiveness to the plant extract. The phosphorylation of AMP-activated protein kinase (AMPK), was also increased in both cells. AMPK is an important regulator of cellular energy homeostasis and a proven therapeutic target for metabolic disorders such as type 2 diabetes (Kim et al., 2016; Aydin et al., 2025). Activation of AMPK stimulates glucose uptake and fatty acid oxidation while inhibiting lipogenesis and gluconeogenesis, which is similar to the mechanism of action of metformin (Ke et al., 2018; O'Neill, 2013; Hasanvand, 2022).

Similarly, activation of the PI3K/Akt signalling pathway, a central pathway in the regulation of glucose homeostasis by the ethanolic extract of GP attenuates insulin resistance and restores glucose uptake (Guo S. et al., 2021; Fontana et al., 2024). Gene expression analysis revealed an upregulation of the Akt, PI3K and GLUT4 genes and a significant downregulation of the GS and GSK 3β genes. These gene findings were supported by protein analysis, which revealed higher levels of PI3K, Akt, p-GSK 3β, p-Akt, GLUT4, p-GS and p-PI3K in the GP-treated group compared to the control group. Conversely, GS and GSK 3β protein expression showed remarkable downregulation. The study showed a significant improvement in glucose content and glucose uptake after treatment with different concentrations of GP. This dose-dependent improvement emphasises the therapeutic potential of GP in improving insulin resistance and promoting effective glucose utilisation. Overall, these molecular and biochemical findings highlight the ability of the ethanolic extract of GP to modulate the PI3K/Akt signalling pathway and associated downstream targets, underpinning its role as a natural candidate for the treatment of insulin resistance and hyperglycaemia (Feng et al., 2024).

In addition, previous studies have shown that the antihyperglycaemic effects of GP may be mediated by the modulation of key enzymes involved in insulin signalling pathways, in particular glycogen synthase kinase three beta (GSK3β). Kaempferol, a bioactive flavonoid metabolites of GP, is thought to be involved in this mechanism and exerts an inhibitory effect on GSK3β activity (Wong et al., 2015; Yang et al., 2022). The study showed that HepG2 cells treated with the aqueous extract of GP exhibited increased phosphorylation of GSK3β (Ser9), which in turn led to an increase in glucose, while dysregulation of GSK3 activity is associated with insulin resistance and hyperglycaemia (Henriksen, 2010; Liu and Yao, 2016). GP was also found to increase glucose uptake in hyperglycaemic HepG2 cells in a concentration-dependent manner, highlighting its potential as a natural therapeutic agent to control elevated blood glucose levels.

In contrast, a study conducted with β-cells in the islets of Langerhans of T1DM rats showed no improvement in cell viability after administration of GP water extract (Hassan et al., 2010). However, remarkable changes were observed in the distribution pattern of insulin-positive cells in the pancreatic tissue of T1DM rats, with a significant decrease in the number of insulin-positive cells. This decrease is consistent with the autoimmune destruction of β-cells that characterises T1DM and suggests that GP extract has no protective or regenerative effects on pancreatic insulin-secreting cells (Roep et al., 2021; Atkinson and Mirmira, 2023). Treatment of RIN-5F cells, the cloned pancreatic β-cells, with different concentrations of GP water extract also did not lead to a significant increase in insulin levels or an improvement in cell viability. This indicates that the hypoglycaemic effect of the extract is not dependent on insulin secretion. The study concluded that the ability of GP to enhance or mimic insulin action at the cellular level may be related to the insulin-like properties of the active ingredient contained in the extract. Therefore, further studies are needed to identify the active ingredient in GP extract that may play a role in these insulin-like properties which could contribute to the development of new therapies to combat insulin resistance.

### 4.2 *In vivo* studies

Several *in vivo* studies have demonstrated the beneficial blood glucose-lowering effect of DM, which is consistent with traditional claims of its therapeutic use and indicates its potential for diabetes management. For example, repeated oral administration of 250 mg/kg body weight/day of the methanolic extract and its various soluble fractions (SF), including aqueous (AQSF), chloroform (CSF), ethyl acetate (EASF), and petroleum ether (PESF), was found to lower blood glucose levels in T1DM rats for 21 days. AQSF, CSF and PESF showed a greater decrease in blood glucose levels than the other fractions (Jobaer et al., 2023). Similarly, in a 28-day dietary experiment in T1DM rats, administration of dry GP leaf powder was shown to decrease blood glucose levels, increase body weight, decrease triglycerides, cholesterol and low-density lipoproteins (LDL), and increase high-density lipoproteins (HDL) (Nath et al., 2023). These changes reflect the lipid-lowering and cardioprotective potential of GP, which is particularly beneficial for diabetics, who are at increased risk of cardiovascular complications. Furthermore, in a T2DM model using eight-week-old male C57BL/6JJL mice fed a high-fat diet (HFD), administration of dried GP powder resulted in a significant reduction in fasting and 2-h blood glucose levels in HFD mice after 3 months and maintained this effect for up to 5 months (Aung et al., 2021).

A previous study has shown that administration of the ethanolic extract of GP at different doses (500, 750 and 1,000 mg/kg) over a treatment period of approximately 42 days led to an increase in body weight in rats with T1DM and showed dose-dependent hypoglycaemic effects (Tahsin et al., 2022). In rats receiving higher doses, the reduction in blood glucose levels was more pronounced, suggesting that the antihyperglycaemic effect of GP is concentration-dependent and possibly related to the better bioavailability of the active phytochemicals at higher doses (Alfahel et al., 2023). Similarly, repeated administration of 50, 150 and 300 mg/kg body weight of an ethanolic GP extract to T1DM rats over a 7-day period resulted in an increase in body weight, a decrease in serum total cholesterol levels and a decrease in triglycerides in these rats. This indicates that GP is able to improve glucose tolerance in STZ-induced T1DM rats but not in normal rats (Zhang and Tan, 2000). The improvement in glucose tolerance could be due to an improvement in insulin sensitivity or modulation of hepatic glucose production and the antidiabetic activity of GP could be more strongly activated under hyperglycaemic conditions. (Li et al., 2022).

The study on the effect of GP also shows acute antihyperglycaemic activity and 14-day antihyperglycaemic activity in T1DM rats. This study showed a significant reduction in blood glucose levels of 25% ethanol extract (EE) and all GP fractions (ethyl acetate fraction (EAF), n-butanol fraction (n-BF) and aqueous fraction (AF)), with the EAF significantly lowering blood glucose levels at 3 and 5 h (Algariri et al., 2014). Further analysis showed that the n-BF and AF fractions of GP exhibited antihyperglycaemic effects comparable to those of metformin, suggesting that it could affect glucose metabolism via similar pathways such as modulating AMPK activity, altering insulin signalling or increasing the expression of glucose transporters (Entezari et al., 2022; Lee et al., 2022). In addition, the 14-day antihyperglycaemic activity showed a significant reduction in blood glucose levels, with the n-BF fraction showing the most potent effect. The efficacy of this fraction could be related to the presence of flavonoids and other phytoconstituents known for their insulin-mimetic or insulin-sensitising properties (Vinayagam and Xu, 2015; Zanzabil et al., 2023).

Meanwhile, Hassan et al. (2010) showed a significant reduction in body weight and fasting blood glucose levels as well as improved glucose tolerance and marked improvement in glucose utilisation 15–120 min after glucose loading in T1DM rats after 14-day of administration of 500 or 1,000 mg/kg GP water extract (Hassan et al., 2010). In addition, the isolated abdominal muscle of T1DM rats showed a significant increase in glucose uptake. This finding suggests a peripheral mechanism of action likely mediated by increased glucose transporter activity or enhanced intracellular glucose metabolism in skeletal muscle, which plays an important role in glucose utilisation (Chang et al., 2004; Chadt and Al-Hasani, 2020). The same study also concluded that GP inhibits endogenous insulin production and does not stimulate insulin secretion. In another study using different fractions of GP (n-butanol, hexane and ethyl acetate), a significant decrease in blood glucose levels was observed in T1DM rats, with the ethyl acetate fraction showing the most significant effect compared to the other fractions (June et al., 2012). The study also found that GP inhibits GSK3β, suggesting that the hypoglycaemic effect of the GP fractions may be due to direct or indirect effects on the activities of one or more components upstream of the insulin signalling pathway.

GP has also been reported to have an insulinomimetic effect due to its high content of flavonoids and glycosides. GP has been found to inhibit gluconeogenesis in the liver, stimulate glycogenesis, stimulate hepatic glucose and lower hepatic endogenous glucose (Lee et al., 2012; Sok Yen et al., 2021). This contributes to better glycaemic control. In a study on the T1DM rat model, administration of ethanolic and aqueous extracts of GP led to an increase in liver glycogen content, but not to an increase in plasma insulin concentration (Lee et al., 2012). Interestingly, there was a decrease in fasting blood glucose and HbA1c levels. These findings suggest that the glucose-lowering and glycogen-promoting effect of GP is not mediated by increased insulin secretion, but may be due to insulin-independent mechanisms (Panahi et al., 2020). Thus, the ethanolic extract has the potential as an adjunct treatment in the treatment of DM due to its antidiabetic effect, which is comparable to that of metformin. However, further studies are needed to investigate the mechanism of action, long-term efficacy and safety profile.

On the other hand, in-depth studies at the molecular level revealed several potential signalling pathways associated with the role of GP in alleviating T2DM. The ethanolic extract of GP was found to strongly affect key proteins and markers in the two disease-related protein signalling pathways, such as phosphoinositide 3-kinase/protein kinase B (PI3K/AKT) and receptor for advanced glycation end products (AGE-RAGE) (Guo W. et al., 2021). Genetic analysis of the study using the T2DM rat model revealed that the genes for eNOS, AKT, MAPK and iNS were significantly upregulated in the GP group compared to the model group, while the genes for caspase-8 and caspase-3 were downregulated. This study also showed the involvement of two other metabolic pathways: retinol metabolism and glycerol phosphate metabolism. Further genetic and protein analyses in the T2DM rat model also showed the effect of the ethanolic extract of GP in upregulating GLUT4, Akt and PI3K genes by GP, along with downregulation of GS and GSK 3β genes (Guo S. et al., 2021). This suggest that GP promotes glycogen synthesis and facilitates glucose uptake via insulin-mimetic or insulin-sensitising mechanisms (El-Ashmawy et al., 2022).

**FIGURE 1:**
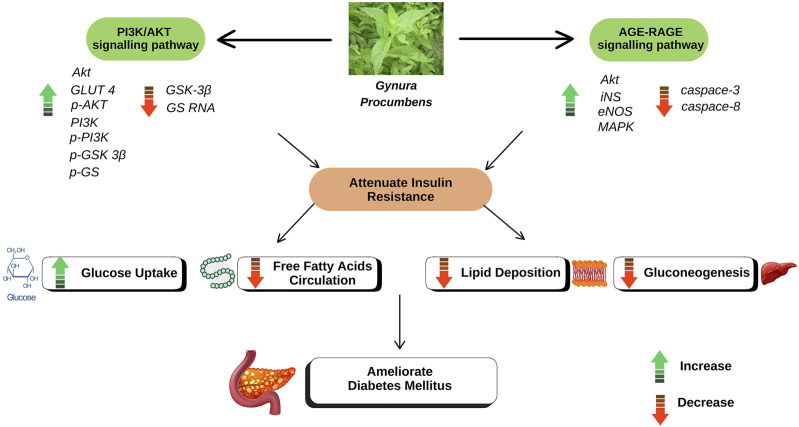
The proposed mechanism of action of GP in ameliorating DM.

In particular, RAGE, a molecule from the immunoglobulin superfamily, acts as a receptor for advanced glycation end products (AGEs) (Ong et al., 2018). AGEs are formed by non-enzymatic glycation and protein oxidation, especially in the presence of elevated blood glucose levels (Twarda-Clapa et al., 2022). Consequently, RAGE is considered a key player in the accumulation of various ligands in diabetic tissues (Ramasamy et al., 2011). In addition, PI3K/Akt also plays an important role in cell physiology by regulating the transmission of growth factor signals during important cellular processes and organismal growth, including glucose homeostasis and metabolism (Huang et al., 2018; Savova et al., 2023). The PI3K/AKT signalling pathway controls lipid and glucose metabolism under the guidance of insulin. Under normal circumstances, insulin is released immediately after food intake, which triggers activation of the PI3K/AKT signalling pathway. In T2DM, however, there is a reduced response to insulin which disrupts the activity of the PI3K/AKT signalling pathway. This can lead to reduced insulin secretion by the pancreas, impaired glucose utilisation, increased release of free fatty acids in adipose tissue, decreased lipid accumulation in the body, increased gluconeogenesis in the liver and muscles, and a loss of fine regulation of lipid and glucose metabolism (Huang et al., 2018; Petersen and Shulman, 2018; Feng et al., 2024). A summary of the antidiabetic properties of GP can be found in Table 3 and the proposed mechanism of action of GP is shown in Figure 1.

**TABLE 3:** Summary of the effects of GP on DM.

Type of model	Treatment dosage	Treatment duration	Findings	References
*In vitro* studies				
C2C12 muscle cells 3T3-L1 adipocytes	GP water extract (100 μg/mL) and ethanolic extract (100 μg/mL)	24 h	↑ AMPK phosphorylation in both cells Water extract induced GLUT4 membrane translocation in 3T3-L1 adipocytes Both extracts induced GLUT4 membrane translocation in C2C12 muscle cells GLUT4 membrane translocation was more prominently in the C2C12 cells than in 3T3-L1 cells	Aung et al. (2021)
HepG2 cells	0.0391–1.2500 mg/mL ethanolic extract	24 h	↑ Akt, PI3K and GLUT4 genes ↓ GS and GSK 3β genes ↑ PI3K, Akt, p-GSK 3β, p-Akt, GLUT4, p-GS, and p-PI3K proteins ↓ GS and GSK 3β proteins Regulated glucose metabolism and insulin resistance via PI3K/Akt signalling pathway	Guo et al. (2021a)
HepG2 cells	0.1 μg/mL aqueous extract	24 h	↑ glucose consumption by 51% ↑ phosphorylations of GSK3β	Wong et al. (2015)
β-cells in the islet of Langerhans of diabetic rats Insulin-positive cells in the pancreas of diabetic rats RIN-5F cells	1, 5 or 10 mg/mL water extract	72 h 72 h 4–5 days for insulin secretion	No improvement in β-cells viability ↓ insulin-positive cells ↑ insulin levels in RIN-5F cells ↑ RIN-5F cells viability	Hassan et al. (2010)
*In vivo* studies				
Wistar albino rats induced T1DM by 120–150 mg/kg alloxan	250 mg/kg BW/day methanolic extract and its soluble fractions: AQSF, CSF, EASF and PESF	21 days	↓ blood glucose level AQSF, CSF and PESF showed a greater reduction in glucose levels compared to the other fractions	Jobaer et al. (2023)
Female Swiss albino mice induced T1DM by 150 mg/kg alloxan monohydrate	0.5% and 1.0% dry GP leaf powder	28 days	↓ glucose level ↑ body weight ↑ HDL level ↓ triglycerides, cholesterol, and LDL levels	Nath et al. (2023)
Male C57BL/6JJL mice induced T2DM by fed with a high-fat diet (HFD-60)	1% dried GP powder	1, 3 and 5 months	↓ fasting and 2-h blood glucose levels	Aung et al. (2021)
Adult male Wistar rats induced T1DM with 150 mg/kg alloxan	500, 750 and 1,000 mg/kg BW/day ethanolic extract	42 days	Hypoglycemic activities in a dose-dependent manner ↑ body weight	Tahsin et al. (2022)
Male Sprague-Dawley induced T1DM by 60 mg/kg body weight STZ	50, 150 and 300 mg/kg BW/day ethanolic extract	7 days	↑ body weight ↓ serum glucose level ↓ triglyceride ↓ serum cholesterol level	Zhang and Tan (2000)
Male and female Sprague Dawley rat, induced T1DM by 55 mg/kg STZ	Acute antihyperglycemic activity: 500, 1,000 and 2000 mg/kg BW/day of 25% EE, EAF, n-BF and AF 14-day period antihyperglycemic activity: 500 and 1,000 mg/kg BW/day of 25% EE, EAF, n-BF and AF	14 days in total	Acute antihyperglycemic activity ↑ body weight, but insignificant compared to the control group EAF fraction ↓ blood glucose level comparable to metformin after 3 and 5 h 14-day period antihyperglycemic activity ↑ body weight ↓ glucose levels n-BF fraction showed the highest efficacy in reducing glucose levels in a dose-dependent manner	Algariri et al. (2014)
Sprague Dawley rats induced T1DM by 55 mg/kg STZ	0, 25, 50, 75% and 95% (ethanol in water)	Up to 120 min, 7 h and 14 days for each treatment	↑ body weight after 14 days 95%, 25% and 0% extracts exerted significant effects on blood glucose from 15 to 120 min 25% ethanol extract demonstrated the most fasting blood glucose-lowering effect in acute/single oral administration and the 14-day study	Algariri et al. (2013)
Male Sprague-Dawley rats, induced T1DM by 65 mg/kg body weight STZ	500 or 1,000 mg/kg BW/day of water extract	14 days	↓ body weight ↓ fasting blood glucose level ↑ glucose tolerance ↑ glucose disposal ↑ muscle glucose uptake No glucose absorption	Hassan et al. (2010)
Male Sprague-Dawley rats induced T1DM by 45 mg/kg body weight STZ (i.v)	Hexane, ethyl acetate and n-butanol fractions at 250 mg/kg BW/day	2 weeks	↓ blood glucose level ↑ liver glycogen contents GSK3β was phosphorylated	June et al. (2012)
C57BL/KsJ-db/db mice (db/db mice were derived from autosomal recessive inheritance of an C57BL/KsJ inbred strain used as T2DM model)	3 g/kg BW/day ethanolic extract	5 weeks	↑ protein expression of AKT, eNOS, iNS and MAPK ↓ expression of caspase-8 and caspase-3 ameliorated insulin resistance and sensitivity via PI3K/Akt and AGE-RAGE signalling pathway	Guo et al. (2021b)
Male and female C57BL/KsJ db/db mice (used as T2DM model)	3 g/kg BW/day ethanolic extract	5 weeks	↓ water and food intake ↑ body weight ↓ blood glucose levels after 2 h and fasting blood glucose level ↓ serum TC and TG levels ameliorated insulin resistance and sensitivity via PI3K/Akt signalling pathway	Guo et al. (2021a)
Male Sprague Dawley rats induced T1DM by 55 mg/kg body weight STZ	50, 100 and 150 mg/kg BW/day aqueous and ethanolic extract	42 days	↓ HbA1c and fasting blood glucose levels ↑ liver glycogen content Ethanolic extract showed better improvement than aqueous extract in a dose-independent manner and comparable to metformin Liver hexokinase activity ↑ in 100 mg/kg body weight of aqueous extract ↓ in 100 and 150 mg/kg body weight of ethanolic extract Liver phosphofructokinase activity ↑ in 100 and 150 mg/kg body weight of ethanolic extract led ↑ in 100 mg/kg body weight of aqueous extract Liver fructose-1,6-bisphosphatase activity ↓ in all dosages of aqueous extract showed a decrease in diabetic rats ↓ in 100 and 150 mg/kg body weight of ethanolic extract	Lee et al. (2012)

## 5 Safety of *Gynura procumbens* (Lour.) Merr

Preclinical studies have generally shown that GP is safe for consumption at various doses. For example, the study by Algariri et al. (2014) showed that the maximum dose of the extract of 2000 mg/kg in an acute toxicity test did not result in any treatment-related mortality during the 14-day observation period (Algariri et al., 2014). In addition, the acceptable daily intake (ADI) was set at 700 mg/kg/day. At the same time, the growth rate and indices of liver, kidney and haematopoietic function were also unaffected, indicating that the extract is safe and no acute toxicity was observed, as the LD50 for female rats is above 2000 mg/kg.

Furthermore, administration of 2 and 4 g/kg GP to rats in a 14-day toxicity trial did not cause any abnormalities in serum biochemical parameters (liver and kidney) or the structure of their organ tissues, with a zero-mortality rate even after the experimental period (Jabbar et al., 2023). The possibility of safe therapeutic use is supported by the absence of adverse effects on organ function, biochemical parameters and histological structures. These results may indicate that GP is a safe candidate of complementary therapy for the treatment of DM, as it has a wide safety margin. Despite the encouraging preclinical results, long-term toxicity, reproductive safety and human clinical studies are still scarce. To ensure the safety of the extract for long-term use in DM, these factors still need to be thoroughly investigated.

## 6 Future direction

Despite these encouraging data on the potential antidiabetic properties of GP, the evidence for its efficacy in the treatment of DM is still inconsistent. Future experimental research should focus on the testing of plant extracts for which comprehensive phytochemical fingerprints should be established according to the ConPhyMP guidelines to improve reproducibility and transparency in phytochemical pharmacology (Heinrich et al., 2022). This includes comprehensive taxonomic authentication using voucher specimens, comprehensive reporting of collection and extraction conditions, and the use of more than one orthogonal analytical technique, such as UHPLC-QTOF-MS/MS, HPLCMS, GCMS and HPTLC, to generate robust chemical fingerprints.

In addition, the lack of standardisation of the extract preparation can lead to different concentrations of the bioactive metabolites, which poses a major challenge for reproducibility and efficacy. For example, aqueous extracts can have different pharmacological effects compared to ethanolic extracts due to differences in solubility and stability of the active metabolites. Thus, standardising the extraction method and ensuring a consistent phytochemical profile is crucial to improve the reproducibility of results and demonstrate the clinical utility of GP in the treatment of DM.

In addition, the bioactive compound responsible for the observed effects was not clearly identified and quantified in some studies. The different study designs and the lack of clinical trials may also contribute to some inconsistencies and limit the generalisability of the results. To clarify these issues, more comprehensive studies are needed to identify the bioactive compounds and molecular targets as well as the mechanisms of action and to assess long-term safety. Potential risks that may be associated with prolonged use of GP, such as hepatotoxicity, nephrotoxicity and other systemic effects, also need to be carefully assessed. An understanding of the pharmacokinetics and pharmacodynamics of GP will also support its integration into current treatment regimens.

However, studies on the safety and efficacy, dose and long-term effects of GP in humans in clinical trials are very limited. Most current studies are conducted to investigate the underlying mechanisms and pharmacodynamics in controlled laboratory settings that have not yet been translated to the clinical. Accordingly, the lack of comprehensive clinical data on this product has greatly hindered its inclusion in evidence-based medical systems and practises. Therefore, clinical studies need to be conducted to assess safety, tolerability and dose in healthy humans. This will allow the identification of relevant biomarkers that should be useful not only for monitoring the efficacy of the therapy, but also for the early detection of signs of toxicity. These studies should also include long-term use to detect any chronic toxicities or late-onset adverse effects.

## 7 Conclusion

GP shows promising potential as an antidiabetic agent and has an effect comparable to metformin. By targeting the AGE-RAGE and PI3K/AKT signalling pathways, GP could help to reduce insulin resistance and increase insulin production. However, the evidence remains mixed, as other studies have presented varying results on the efficacy of GP in the treatment of diabetes. This could be due to the lack of standardisation of the extract preparation, insufficient information on the bioactive substance responsible for the observed effects and the lack of clinical studies. Therefore, more comprehensive studies including clinical trials are needed to clarify the discrepancies in the findings and provide a clearer effect of GP in alleviating DM. With these improvements, GP could complement standard DM treatments and offer patients a safer, more holistic approach.
